# Comparison of Three Real-Time Measurement Methods for Airborne Ultrafine Particles in the Silicon Alloy Industry

**DOI:** 10.3390/ijerph13090871

**Published:** 2016-09-01

**Authors:** Ida Teresia Kero, Rikke Bramming Jørgensen

**Affiliations:** 1 Department of Industrial Process, Technology SINTEF Materials and Chemistry, P.O. Box. 4760, NO-7465 Trondheim, Norway; 2 Department of Industrial Economics and Technology Management, Norwegian University of Science and Technology, Trondheim, Norway; rikke.jorgensen@iot.ntnu.no

**Keywords:** electric arc furnace tapping, silica fume, airborne particulate matter, aerodynamic diameter, mobility diameter, electric low pressure impactor, fast mobility particle sizer, condensation particle counter

## Abstract

The aim of this study was to compare the applicability and the correlation between three commercially available instruments capable of detection, quantification, and characterization of ultrafine airborne particulate matter in the industrial setting of a tapping area in a silicon alloy production plant. The number concentration of ultrafine particles was evaluated using an Electric Low Pressure Impactor (ELPI^TM^), a Fast Mobility Particle Sizer (FMPS^TM^), and a Condensation Particle Counter (CPC). The results are discussed in terms of particle size distribution and temporal variations linked to process operations. The instruments show excellent temporal covariation and the correlation between the FMPS and ELPI is good. The advantage of the FMPS is the excellent time- and size resolution of the results. The main advantage of the ELPI is the possibility to collect size-fractionated samples of the dust for subsequent analysis by, for example, electron microscopy. The CPC does not provide information about the particle size distribution and its correlation to the other two instruments is somewhat poor. Nonetheless, the CPC gives basic, real-time information about the ultrafine particle concentration and can therefore be used for source identification.

## 1. Introduction

The modern metallurgical industry is subject to extensive health, safety, and environmental regulations. The topic of airborne particulate matter (PM) is important both in terms of protecting the environment and the health of workers. The industry is directing a great deal of effort and investment towards improvements in technical process changes to reduce exposure, e.g., enclosure respiratory protection, ventilation, and filter systems. These are often site-specifically designed to collect and control the off-gases and dust from the various process operations. Still, it is difficult to collect all the dust and staff exposure can, at times, be high. Lately, studies have indicated the possible presence of ultrafine dust in ferroalloy (including silicon alloys) production [1,2,3].

Epidemiological and toxicological studies have shown that exposure to ultrafine particles (UFP) are associated with negative pulmonary and cardiovascular health effects, especially related to freshly generated ultrafine particles [4,5,6]. Epidemiological studies are performed mainly in relation to ambient air pollution (traffic generated particles), while the toxicological studies are performed with particles common also in occupational atmospheres. It is well known that ultrafine particles are generated in workplaces [7,8]. However, epidemiological studies of the exposure to ultrafine particles in workplace atmospheres are not found. The link between negative pulmonary and cardiovascular health effects and exposure to particulate matter in occupational environments is well established for certain particle fractions, including fine particles (PM_2.5_), which also include the ultrafine fraction [9,10].

For the silicon alloy industry, respiratory symptoms are well known [11,12,13], and recently asthma and chronic obstructive pulmonary disease (COPD) have been associated to exposure in this industry [14]. The industry needs to adhere to the occupational exposure limits (OELs), however the cause-effect relationship between the exposure and the negative health effects for the silicon alloy worker is still not fully understood [15]. Exposure to ultrafine and/or sub-micrometre particles might be a part of the explanation, considering that these particles are often produced during hot processes [7,16,17].

As pointed out by Søyseth et al. [15], dust reduction in the smelting industry should have a high priority to help prevent obstructive lung disease in workers. Ultrafine particles are found in many metal producing industries, including the ferroalloy [2] and aluminium smelters [18]. The silicon alloy industry is often included in the studies performed in the ferroalloy industry but is rarely considered as a separate industry. This is a weakness in these studies, because the dust types in the different industrial branches may be very different. Even dust originating from different processes in the same plant may be complex, diverse, and varied over time. In order to prevent exposure to ultrafine and submicrometer particles, the processes involved in the production of these particles need to be identified, the concentration levels need to be established, and the knowledge about the size distribution of the particles, and how it varies, is required. A suitable method for measuring the ultrafine and submicrometer particles in a hot and polluted atmosphere is essential.

Several measurement techniques can be used to characterize and quantify the ultrafine airborne particulate matter. None of the commercially available instruments are, however, tested under the relatively demanding conditions of an industrial melting plant. Nor are they validated with respect to the sometimes complex characteristics of real, industrial PM. This constitutes a major hurdle, which cannot be overcome by testing or validating the instruments in controlled laboratory settings. The measurements reported on in this paper were carried out by means of an Electric Low Pressure Impactor (ELPI™ by Dekati Ltd., Tampere, Finland), a Fast Mobility Particle Sizer (FMPS^TM^ 3091 by TSI, Shoreview, MN, USA), and a Condensation Particle Counter (CPC 3007 by TSI). The three instruments are all commercially available and offer the possibility to detect particles with aerodynamic diameters as small as 7, 5.6, and 10 nm, respectively. This paper aims to demonstrate how these three instruments can be used to assess PM concentrations in a silicon alloy production plant. Special attention is directed to the instruments’ abilities to detect, quantify, and characterize the concentration of ultrafine particles.

## 2. Silicon Alloy Production

Silicon and ferrosilicon are produced in electric arc furnaces (EAF). Raw materials include crushed or pre-sintered minerals and ores (typically quartz), in addition to carbonaceous reductants (coke and coal). The raw materials are fed at the top of the furnace. Inside the furnace, the raw materials react and form an alloy which is tapped from the furnace into a transportable ladle. The tapped alloy is generally refined in the ladle, by a slag process [19]. The refined metal is then cast and crushed to the customers’ specification. A thorough description of the production processes for silicon rich alloys can be found in the textbook by Schei [20]. The major source of fumes is the furnace itself, yet modern furnace design in combination with powerful ventilation and filter systems are efficient in the removal of most of the furnace top gases and fumes. The collected PM, often referred to as Condensed Silica Fume (CSF), is sold as a valuable by-product. Typically, the tapping, refining, and casting operations are the main sources of fumes inside the plant [21].

The term *“metallurgical fume”*, as used in this paper, designates airborne particulate matter (PM) formed by the evaporation and/or oxidation of liquid metal. When liquid silicon is exposed to air, it oxidizes by a two-step mechanism where the silicon first reacts to form SiO gas, which then oxidizes further into SiO_2_ particles. Naess et al. [3,22,23] provide detailed descriptions of the PM generation mechanisms for silicon alloys. The metallurgical fume generated in the silicon production industry has been characterized by several authors [2,3,24,25]. The CSF protoparticles are typically amorphous silica spheres which may occur independently but more often as agglomerates. In the literature, terms like agglomerate, aggregate, and primary particles are not always unambiguous. We have chosen to adhere to the terminology used by Dingsøyr et al. [24] using the terms protoparticles, primary agglomerates, and secondary agglomerates to describe this type of PM. Primary agglomerates are held together by strong material bridges, whereas secondary agglomerates are held together by weak forces. Næss et al. [3] studied CSF from the tapping and refining processes of metallurgical grade silicon and reported an average protoparticle size of approximately 0.06 µm. Kero et al. [2] used an ELPI to characterize the PM in a tapping area of a ferrosilicon furnace and found an average primary agglomerate size of 0.17 µm. Both studies characterized the fumes as amorphous silica. Dingsøyr et al. [24] and Kolderup [25] reported the characteristics of fumes from the furnace charge top, which may be less relevant a comparison to the tapping fume studied here. All authors emphasize the influence of furnace operation and process parameters on the size and agglomeration status of the PM collected. Furthermore, the method of size determination will also influence the size estimate, and direct comparisons are therefore not recommended. The ELPI study by Kero et al. [2] uses, for example, a non-uniform density of the particles.

## 3. Experimental Section

Fumes from the tapping area near an electric arc furnace for silicon alloy production were collected and analysed using an ELPI, a FMPS, and a CPC. The measurements were performed during a two day period. The three instruments were sampling synchronously, placed side by side. Samples collected by ELPI were analysed by transmission electron microscopy (TEM; JEOL JEM-2010) equipped with energy dispersive X-ray spectroscopy (EDS).

**Figure 1:**
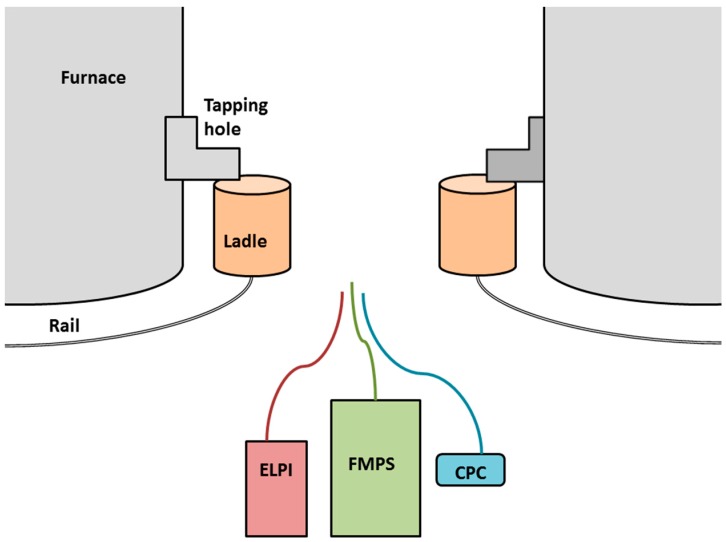
Schematic illustration of the placement of the instruments in the furnace tapping area between two electric arc furnaces. Both furnaces were continuously tapped.

The instruments were set up approximately 15 m from the ladles in the furnace tapping area between two continuously tapped furnaces; Figure 1 shows a schematic illustration of the instruments’ position. In the large hall behind the two furnaces, other PM-generating operations such as refining and casting took place.

### 3.1. The Electrical Low Pressure Impactor (ELPI™)

The ELPI (model 972E, Dekati Ltd., Tampere, Finland) classifies aerosols according to their aerodynamic diameter, *D_p_*, and collects real-time particle size measurements in the size range of 7 nm–10 µm. These stages and their aerodynamic diameter intervals with corresponding geometric mean diameter (GMAD) values are detailed in Table 1.

The operating principle of the ELPI includes particle charging in a Corona charger; inertial classification in a cascade impactor; and electrical detection of the collected particles by a multichannel electrometer, which are all integrated parts of the ELPI. An external vacuum pump is used to control the airflow (10 L/min) through the instrument.

The particles enter the ELPI by use of the vacuum pump. They pass through the charger unit where the particles become electrically charged. The particles then enter the impactor unit where they are separated into 12 different stages depending on their aerodynamic behaviour. Each impactor stage is connected to an electrometer. When a charged particle impacts on a substrate it produces an electrical current, recorded by the electrometer (Note that the impactor classifies the particles according to their flow inertia, not their charge). A more detailed description of the ELPI function and its principles of operation can be found in the literature [26,27,28,29,30].

A particle size distribution was established based on the size fractions of the impactor stages. A 0.56 m long, flexible tube was attached to the aerosol inlet. The aerosols analysed in this study were collected on greased aluminium foil substrates from which material was transferred onto holey carbon copper grids for transmission electron microscopy. Zeroing was carried out prior to each measurement and electrometer values were checked to be stable after each measurement.

### 3.2. The Fast Mobility Particle Sizer (FMPS^TM^)

A TSI 3091 Fast Mobility Particle Sizer (FMPS, TSI, Shoreview, MN, USA) was used to measure particle size distribution and the particle number concentration in the size range of 5.6–560 nm, divided into 32 size classes. The FMPS was equipped with a 1 µm cyclone, and the flow rate through the instrument was 10 L/min. Particle classification and counting are performed simultaneously through aerosol electrometers, with a time resolution of one second.

The FMPS uses two consecutive unipolar corona chargers of opposite polarity to obtain a predictable charge distribution. The instrument continuously draws an aerosol sample into the inlet. After passing a 1 µm cyclone, the aerosol passes through the charging region where it receives a predictable charge. Net positive charged particles are then introduced to the measurement region near the centre of a high-voltage electrode column and transported down the column via HEPA-filtered sheath air. A positive voltage applied to the electrode creates an electric field that repels the particles outwards according to their electrical mobility. Charged particles strike the respective electrometers and transfer their charge. A particle with high electrical mobility strikes the electrometer near the top, whereas a particle with lower electrical mobility strikes an electrometer lower in the stack. This arrangement, using highly sensitive electrometers, allows for concentration measurements of multiple particle sizes simultaneously. In order to avoid the build-up of PM on the charger needles of the FMPS, the instrument was coupled to a dilutor, with a dilution rate of 100:1 (DIL 550, TOPAS GmbH, Dresden, Germany). The measurements were performed by use of a 0.59 m long flexible conductive silicone tube.

The FMPS spectrometer performs particle size classification based the mobility diameter (D_m_). The size distribution of the particles is measured in 32 channels; the smallest channel has a midpoint of 6.04 nm and the largest channel has a midpoint of 523.3 nm. On a logarithmic scale, the size width of each channel is equal (0.062818).

Zeroing of the FMPS instrument was performed at start-up on each day. After zeroing, the effect was controlled (zero-check) by use of a HEPA filter. No measurement was started before the zero check was satisfactory.

### 3.3. The Condensation Particle Counter (CPC)

A TSI 3007 Condensation Particle Counter (CPC), (TSI, Shoreview, MN, USA) was used for measurements of the number concentration of particles in the size range of 10 to greater than 1000 nm with a time resolution of 1 s.

The instrument was operating at a flow rate of 0.7 L/min. The sample flow passed a saturator where the air was supersaturated with isopropyl alcohol vapour, followed by a condenser, where the alcohol condensed onto the particles to form droplets which are sufficiently large to scatter the following laser beam. The interruption of the laser beam by the droplets was used to count the particles and to determine the particle number concentration. The CPC detected PM in the range of 10 nm to greater than 1 µm, with a time resolution of 1 s. The maximum concentration is 100,000 particles/cm^3^. The measurement accuracy is ±20% according to the manufacturer's information. The CPC was recently calibrated.

Each measurement period was held for a maximum of 2 h before the CPC was loaded with isopropanol alcohol and restarted.

## 4. Data Analysis

The FMPS measurements were performed by use of the instrument software Fast Mobility Particle Sizer Software version 3.1.1. The software considers the dilutor, and the measurements are automatically recalculated to the correct concentrations. The UFP number concentration is calculated in Microsoft Excel as the sum of particles of the first 20 channels, resulting in the particle concentration of 5.6 nm–100 nm (named *FMPS-UFP*). The total number concentration within the size range of 5.6–560 nm was calculated and named *FMPS*. For the ELPI, only the collection from the size fractions with Dp < 0.6 µm were used for comparison with the other two instruments; this concentration range is henceforth named ELPI. The UFP number concentration was calculated as the sum of the particles of the first three size bins (7–91 nm) and named ELPI-UFP. In the data treatment for ELPI, the particles were assumed to have a uniform density (1 g/cm^3^); this assumption is discussed below. The software used was ELPIvi version 4.0 and Microsoft Excel with a macro supplied by the producer (Dekati Ltd.).

**Figure 2:**
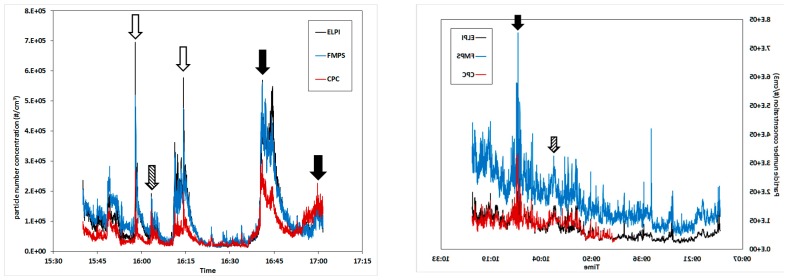
PM concentration as a function of collection time from the two different days of measurement. CPC, FMPS, and ELPI are compared and PM-increasing events are market by arrows. Results from day 1 are shown to the left, day 2 to the right.

At day one, the number of measurements for the FMPS was 4883, and for the CPC and ELPI the number of measurements were 4889. On the second day, the number of measurements for the CPC was 2482, and for the ELPI and FMPS the number of measurements were 4266. Standard measures of central tendency and distributions (arithmetic mean (AM), geometric mean (GM), and geometric standard deviation (GSD)) were calculated for the total particles and for the UFPs. The minimum value (i.e., approximate base line) and the maximum peak value are also reported in Table 2. Note that the table values for day two only include the period where all the instruments ran simultaneously, as opposed to Figure 2 where all data is included.

The measurements are performed with 1 s resolution. To make sure that the measurement series by the three different instruments had the same time settings, time series analyses were performed, and the timelines were adjusted to the optimal time lag. The results were compared in scatterplots, including regression analysis. As recommended by Held et al. [31], the correlation between measurements were evaluated using Spearman’s correlation coefficient.

In order to compare the particle size distributions between the ELPI and the FMPS, particle size distribution is reported as normalised concentration (dN/dlogDp):

$$\frac{dN}{d\;logD_{p}}=\frac{dN}{\log D_{pu}-logD_{pl}}$$

where *dN* is the particle concentration, *D_p_* is the midpoint particle diameter, *D_pu_* is the upper channel diameter, and *D_pl_* is the lower channel diameter.

## 5. Results and Discussion

Figure 2 shows the collection curves of the CPC, FMPS, and ELPI. The arrows mark the events which were identified as the sources of the increased PM concentrations. The process operations which caused the most significant peaks include post-tapping refining (without suction hood; filled, arrows), extended periods of oxygen blowing into the tapping hole (open arrows), and stoking the tapping hole with a wood pole (striped arrows).

The statistical values calculated from the data in Figure 2 are shown in Table 2. The peak (max) values are typically one order of magnitude larger than the baseline (min) values and both the standard deviation and GSM are relatively low [8,32]. The temporal covariation between the three instruments is excellent but the recorded concentrations differ somewhat, most notably for the CPC in comparison with the other two instruments on day 1. On day 2 the FMPS measured noticeably higher concentrations than both the EPLI and CPC. The explanation for the low fit between the CPC and the ELPI/FMPS is related to the measurement ranges. The “max range” of the CPC is 100,000 particle/cm^3^. Above this concentration, coincidence may occur, i.e., two or more particles may be detected as one by the laser beam. The result will be an underestimation of the concentration in industrial settings with high PM load, such as the tapping area. With increasing concentration above this limit, the error will increase. As seen from the results, the instrument is useful for identification of the significant peaks, but not for measuring the correct concentrations.

While all the instruments seemed to detect a continuous increase in PM concentration on day 2, the FMPS recorded a greater increase than the other two instruments, which could be attributed to fouling of the electrometers. Prior to the measurement campaign, the FMPS was validated against a well-known pollution source at the lab and found to be reliable. A zeroing operation was carried out at start-up on each day but a more frequent zeroing of the FMPS electrometers during the measurements series could have potentially prevented this.

### 5.1. Statistical Data Evaluation

**Figure 3:**
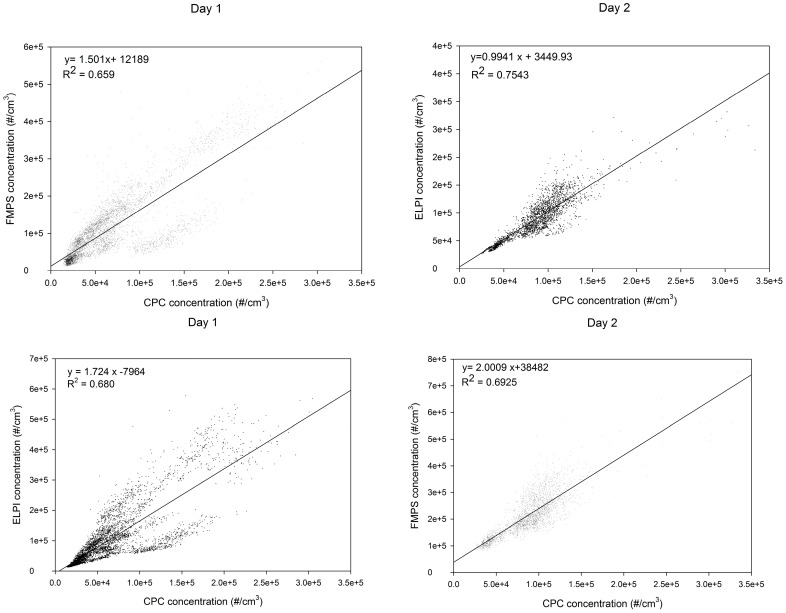

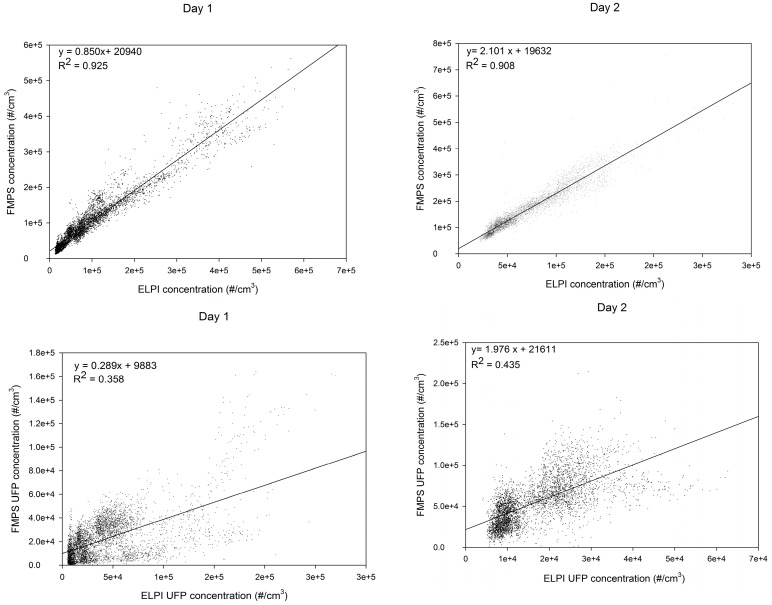
Scatterplots of the particle concentrations of the different instruments together with regression analyses.

Figure 3 shows scatterplots together with regression analyses between the instrument results. The correlation between ELPI and FMPS is very good on day 1, and on day 2 the FMPS seems to measure high concentrations (by a factor of two) as discussed before. Between the ELPI and CPC the best correlation was found on day 2. For the ultrafine fraction of the ELPI and FMPS, the correlation is insufficient, which is further discussed below. The correlations were confirmed by Spearman’s rank correlation coefficient, which showed a correlation of FMPS to ELPI of ρ = 0.959 (Figure 2, left: day 1) and 0.957 (Figure 2, right: day 2), respectively. The correlation between the CPC and the other two instruments is lower; for FMPS to CPC, ρ = 0.814 (day 1) and ρ = 0.666 (day 2). For ELPI to CPC, ρ = 0.882 (day 1) and ρ = 0.862 (day 2) and for ELPI UFP to FMPS UFP, ρ = 0.541 (day 1) and ρ = 0.715 (day 2). ELPI UFP compared to FMPS UFP concentrations are shown in Figure 6.

The results may be compared to the literature, but no comparable results for AM or GM in the smelting industry have been found. Kero et al. [1] found that max peak concentrations were 10 times higher in the ferroalloy industry. Thomassen et al. [18] found comparable max peak concentrations in an aluminium smelter for prebake pot rooms (1.2 × 10^5^ particles/cm^3^) and Søderberg pot rooms (2.5 × 10^5^ particles/cm^3^). However, max peaks that were 100 times higher were recorded during the “change operation” in the prebake pot room. Compared to other types of industries, the results found in this study seems to be quite similar; Evans et al. [33] reported GM values for UFP in a grey iron foundry, ranging from 7.0 × 10^4^ to 2.8 × 10^5^ particles/cm^3^. Cheng et al. [34] found AM and GM values of UFP in an iron foundry to be 7.06 × 10^4^ and 6.14 × 10^4^ particles/cm^3^, respectively. Elihn et al. [35] found comparable median concentrations for aluminium fettling. Kim et al. [36] found GM values in rubber manufacturing, measured by ELPI instrumentation, to be 1.84 × 10^5^ for the entire measuring period, but 5.45 × 10^5^ for the “final process”.

### 5.2. Particle Size Distributions

**Figure 4:**
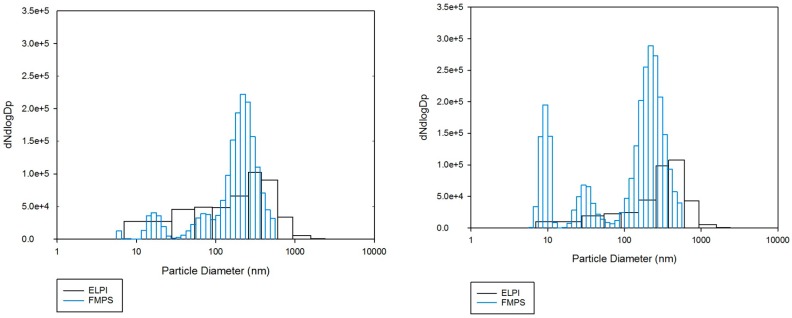
Particle size distributions measured by the ELPI and the FMPS during two different days of measurement. Results from day 1 are shown on the left, day 2 on the right.

The particle size distributions as obtained by the ELPI and the FMPS are shown in Figure 4. The size intervals of the ELPI are much wider than those of the FMPS, which results in a low resolution particle size distribution. The greater resolution of the FMPS is a significant advantage, as it allows detailed information about the size distribution even for ultrafine particles. The FMPS results show a tri-modal particle size distribution, while the ELPI results appear as a simple distribution, with only one mode.

The comparison of results obtained by instruments operating with different measurement principles is non-trivial and quite dubious, as previously discussed by others [37,38]. Leskinen et al. [37] noted that the results obtained by an ELPI and a scanning mobility particle sizer (SMPS) agreed well for spherical particles. They also observed, however, that as the complexity of the particle shapes increase, so does the discrepancy between the instruments. The specific nature of the metallurgical fume studied here, with agglomerates rather than ideal, spherical particles, can at least partly explain the discrepancies. The charging mechanisms are different which may also affect the results and induce more significant differences the more the particle shapes deviate from the ideal shape.

Furthermore, impactors and mobility devices measure different properties, the output parameters being the aerodynamic diameter, D_p_, versus the mobility diameter, D_m_. The key link between these two parameters is the effective density, ρ_p_, which is often unknown. The influence of density on the ELPI results is significant and non-trivial to assess [39,40]. This is because the effective density is difficult to determine experimentally, particularly for ultrafine particles. In addition, the density may vary as a function of the particle size. This is particularly cumbersome if the measurement range is large, as for the ELPI where it covers three orders of magnitude (7 nm–10 µm). Keskinen et al. [41] used a density of 2.2 g/cm^3^ for amorphous silica fume and indicated that it would be valid for particles with D_p_ > 10 nm. Price et al. [38] suggested that 1.3 g/cm^3^ is a better estimate of the effective density of CSF. Since no well-established effective density value for industrial silica fume is available, the unit density was utilized in this study.

### 5.3. Ultrafine Particles

**Figure 5:**
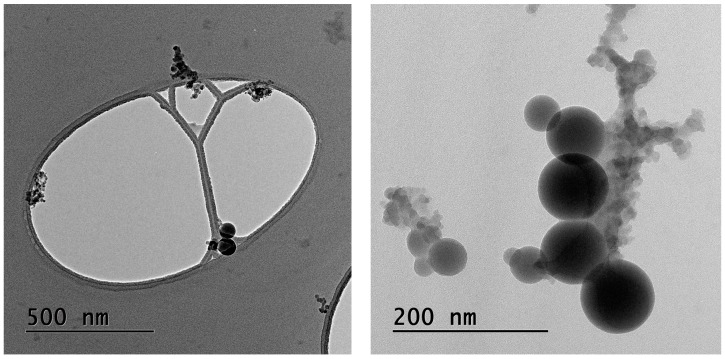
Transmission electron micrographs of ultrafine PM collected by the ELPI.

Figure 5 shows transmission electron micrographs of the PM collected by the ELPI in the second smallest size fraction (D_p_ 28–54 nm). In Figure 5 (left), the magnification is relatively coarse and the holey carbon film of the sample holder is seen as the egg-shaped hole. The particles collected on the film are agglomerates of different shapes and consist of amorphous silica, very similar to findings reported by others [2,3,24,25]. As seen in Figure 5 (right), with larger magnification, the protoparticles are spheres and the transitions between the smallest protoparticles appear blurred, which indicate that they are undergoing a sintering/coalescing process. These agglomerates are therefore likely to be primary agglomerates.

Although the most significant tapping area operations for ultrafine particle generation appear to be post-tapping refining, extended periods of oxygen blowing into the tapping hole, and stoking the tapping hole with a wood pole, it is well-known that any flame can generate ultrafine particles. The ELPI, FMPS, and CPC instruments do not separate between particles of different compositions, however, in the TEM study a number of carbon particles were identified; these are most likely soot from the burning wood pole used for stoking the tapping hole.

The occurrence of primary and secondary agglomerates is important from an occupational hygiene perspective. The ELPI and FMPS both use charging of particles to enable detection. It is, however, possible that the weak forces keeping secondary agglomerates together may be broken in the chargers of the instruments. Indeed, the particles sizes detected here are of the same order of magnitude as the particle sizes reported for primary agglomerates [24]. As human airways and personal respiratory protection do not impart any similar charging, human exposure is more likely to be dominated by the larger secondary agglomerates which are not analysed by these instruments.

**Figure 6:**
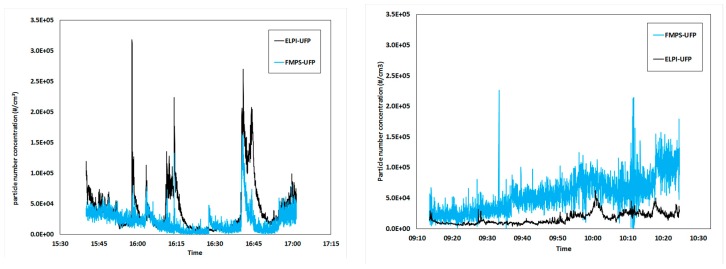
Ultrafine PM concentration as a function of collection time, as recorded by the ELPI and the FMPS during two different days of measurement. Results from day 1 are shown on the left, day 2 on the right.

The collection curves of the ultrafine size fractions as measured by the ELPI and the FMPS are compared in Figure 6. The main source of UFPs in the melting plant appear to be metallurgical fumes from tapping and refining. It should be noted that these measurements were performed relatively close to the source (i.e., the tapping hole) and that the particles may grow and agglomerate during airborne transport. Hence, the concentrations presented here may not be representative for other parts of the same plant. It is hereby acknowledged that a larger number of measurement series, preferably performed in different positions at the same plant, would have been valuable, however this was prevented by practical and economic considerations. Nonetheless, the measurements were long enough to produce an acceptable amount of data and covered most of the processes and operations where UFP may be generated in a silicon alloy smelter.

### 5.4. Instrument Evaluation and Recommendations

The main advantage of the CPC 3007 is that it is small and handheld. It is easy to transport and operate, even in dirty atmospheres. It is the most user-friendly instrument of the three instruments evaluated in this study, and can possibly be fitted into a moderate-sized rucksack for exposure evaluation, but if the instrument needs to be connected to an external dilutor, the advantage of its small size will disappear. Vinzents et al. [42] used this equipment for performing a personal exposure study, by putting the instrument in a rucksack; however this demands the willingness of the operators, knowing that the weight of the instrument is 7.7 kg. The disadvantage of the instrument is that the measurement range is below the highest concentrations reached in this industry. The result will probably be an underestimation of both the mean and peak concentrations. As seen from the results, the instrument is useful for the identification of the significant peaks, but not necessarily for measuring the correct concentrations. Another disadvantage is that it uses liquid (isopropanol), which needs to be refilled every 6–8 h. Used as a battery operated instrument, it has a battery-lifetime of 5 h before the batteries needs to be recharged or changed.

The main advantage of the FMPS is the excellent time- and size resolution of the results. The main advantage of the ELPI is its resilience, the large measurement range of the instrument, and the possibility to collect size-fractionated samples of the PM for subsequent analysis by, for example, electron microscopy. The FMPS and the ELPI are relatively large instruments and while they are moderately complicated to operate, data interpretation is demanding. If operators with insufficient background knowledge undertake the data interpretation, erroneous data and misleading conclusions may easily result.

## 6. Conclusions

The number concentrations of airborne particulate matter have been evaluated in terms of the particle size distribution and the temporal concentration variations, which have been linked to process operations. The three instruments show excellent temporal covariation. The ELPI and the FMPS provided the most detailed results and are therefore the most adequate for research studies.

The advantage of the FMPS is the excellent time- and size resolution of the results. The main advantage of the ELPI is the possibility to collect size-fractionated samples of the PM for subsequent analysis by, for example, electron microscopy. The detection range of the CPC was largely exceeded in the tapping area and it is therefore not suited for concentration assessment in this type of industrial settings. It does nonetheless provide relative, real-time information about the variations in UFP concentration, and can therefore be useful for source identification.

Comparison of instruments based on different measurement principles is always challenging, most particularly so while the correct effective density values for ultrafine particles remain to be elucidated. The difficulties in data interpretation and comparison notwithstanding, both the FMPS and the ELPI are deemed quite suitable for research applications in the silicon alloy industry.

PM reduction in the smelting industry has a high priority, in order to help prevent obstructive lung disease in workers. Post-tapping refining and oxygen blowing into the tapping holes are identified as main sources of UFP in this study. However, each plant must identify their own sources and take action against them.

## Figures and Tables

**Table 1:** Aerodynamic diameter intervals and geometric mean aerodynamic diameters.

Stage No.	D_p_ Range (µm)	GMAD (µm)
1	0.007–0.028	0.02
2	0.028–0.054	0.04
3	0.054–0.091	0.07
4	0.091–0.153	0.12
5	0.153–0.259	0.20
6	0.259–0.379	0.31
7	0.379–0.609	0.48
8	0.609–0.942	0.76
9	0.942–1.59	1.22
10	1.59–2.38	1.95
11	2.38–3.97	3.07
12	3.97–9.85	6.25

**Table 2:** Statistical Results.

		AM ^A^	GM ^B^	Max ^C^	Min ^C^	GSD ^D^
		Particles/cm^3^				
Day 1	CPC	63,831	50,541	304,689	13,829	1.96
	ELPI	102,596	69,518	695,992	12,737	2.43
	FMPS	107,817	80,624	562,120	12,317	2.18
	ELPI UFP	40,670	27,709	318,244	4619	2.43
	FMPS UFP	21,580	14,771	164,504	53	2.65
Day 2	CPC	87,607	81,487	333,801	24,828	1.49
	ELPI	90,542	82,918	281,890	27,376	1.54
	FMPS	214,451	201,640	753,687	82,608	1.44
	ELPI UFP	21,877	19,481	62,686	4019	1.55
	FMPS UFP	70,554	66,416	214,429	1392	1.44

^A^ Arithmetic mean; ^B^ Geometric mean; ^C^ The lowest and highest measurements; ^D^ Geometric standard deviation.
